# Mental Health, Work Productivity, and Quality of Life in People with Severe Haemophilia A Receiving Prophylaxis: Findings from the CHESS Data Platform

**DOI:** 10.1055/a-2658-6151

**Published:** 2025-08-04

**Authors:** Pratima Chowdary, Letizia Polito, Mark Oellerich, Romain Chafaie, Tom Burke, Tom Blenkiron, Enrico Ferri Grazzi

**Affiliations:** 1 Department of Haematology, Katharine Dormandy Haemophilia and Thrombosis Centre, Royal Free London NHS Foundation Trust, Cancer Institute, University College London, London, United Kingdom; 2 Product Development and Strategy, F. Hoffmann-La Roche Ltd., Basel, Switzerland; 3 Health Economics and Outcomes Research, HCD Economics, Daresbury, United Kingdom; 4 Department of Health and Social Care, University of Chester, Chester, United Kingdom

**Keywords:** haemophilia A, severe haemophilia, emicizumab, mental health, work productivity, health-related quality of life, patient-reported outcomes, prophylaxis, burden of illness

## Abstract

**Background:**

Newer therapeutic options for people with severe haemophilia A (PwSHA), in addition to improved clinical and patient-reported outcomes (PROs), have offered more personalised treatment regimens. This analysis explored mental health, work productivity, and health-related quality of life (HRQoL) among PwSHA in Europe receiving a prophylactic treatment regimen.

**Methods:**

The Cost of Haemophilia: a Socio-economic Survey (CHESS) study is a retrospective cross-sectional study of adult men with haemophilia in Europe. We analysed data from CHESS participants with severe haemophilia A and no factor VIII (FVIII) inhibitors who received emicizumab or FVIII replacement prophylaxis. Data are from patient questionnaires and their treating health care providers. This analysis focused on PROs, including productivity and activity impairment via the Work Productivity and Activity Impairment, HRQoL via the EQ-5D-5L, and anxiety via the 7-item General Anxiety Disorder questionnaire (GAD-7) and depression via the 8-item Patient Health Questionnaire (PHQ-8). SHA treatment and clinical characteristics were also collected, including bleeding events, joint health, and chronic pain. All findings were analysed descriptively.

**Results:**

A total of 350 PwSHA met the inclusion criteria, 94 (27%) of whom provided PROs. Most (68%; *n* = 64) were receiving emicizumab (FVIII prophylaxis, 32%; *n* = 30). Clinical characteristics were generally comparable between emicizumab and FVIII prophylaxis groups, including reported chronic pain (63% and 70%) and problem joints (61% and 63%), with on-demand FVIII use for the treatment of breakthrough bleeding events more commonly reported in the FVIII prophylaxis group (34% vs. 56%). Overall, HRQoL showed comparable EQ-5D-5L scores between the treatment groups, with a marginally higher score in the emicizumab group (0.71 vs. 0.69) compared with the FVIII prophylaxis group. Anxiety and depression scores were both numerically lower in the emicizumab group, suggesting a lower burden of disease (anxiety 7-item General Anxiety Disorder questionnaire [GAD-7] mean scores, 6.0 vs. 7.3; depression PHQ-8 mean scores, 6.8 vs. 7.8). Employed PwSHA in the emicizumab group reported a lower impact of SHA on their work impairment (31% vs. 50%), and only 19% (vs. 33%) of the emicizumab group required assistance with daily activities. More PwSHA receiving FVIII prophylaxis reported a negative impact of SHA on their ability to participate in social activities (70% vs. 56%) and on their physical activity (57% vs. 44%).

**Conclusion:**

Patients receiving emicizumab prophylaxis appeared to have more favourable mental health, work productivity, and HRQoL-related outcomes than those receiving FVIII prophylaxis. These findings were observed in the context of comparable clinical characteristics between emicizumab and FVIII prophylaxis despite evidence of a more complex treatment history for the emicizumab group. This analysis has limitations, including a lack of adjustment for confounding factors.

## Introduction

Haemophilia A (HA) is an X-linked bleeding disorder caused by mutations in the *F8* gene that codes for factor VIII (FVIII) protein, an essential cofactor in the coagulation pathway. The clinical phenotype of people with HA (PwHA) is largely governed by the level of residual FVIII expression, where severe HA is classified as FVIII activity less than 1% of wild-type (<1 IU/dL), moderate disease comprises 1 to 5% of wild-type activity, and the mild form is 5 to 40% activity. 1 2 HA affects approximately 1 in 4,000 to 5,000 males across severities, with severe HA prevalence estimated to be approximately 9.5 cases per 100,000 males. 1 2

The clinical burden of HA is characterised in particular by irreversible joint damage, with acute presentation characterised by recurrent haemorrhages, typically in major joints (knees, ankles and elbows) associated with acute pain and swelling. 3 4 5 Repeated bleeding into the articular space can cause chronic synovial inflammation and, over time, progressive joint deterioration. 4 5 This, in turn, may cause substantial chronic pain, movement restrictions and disability with an increased risk of recurrent bleeding, all of which can greatly impact daily functioning and quality of life for PwHA. 6

The primary treatment goals for severe HA are to decrease mortality and minimise clinical sequelae by preventing bleeding episodes with a prophylactic treatment regimen. 7 8 Replacement clotting factor prophylaxis has historically been the cornerstone treatment for preventing bleeding events; however, standard half-life recombinant and plasma-derived replacement factor prophylaxis requires frequent infusions to be effective, imposing a substantial treatment burden on PwHA. 7 8 9 10 11

Recent innovations in HA treatment, such as the introduction of extended half-life (EHL) factor replacement therapies, have sought to ease the treatment burden and to increase protection against bleeds. 8 10 11 Emicizumab, a subcutaneously administered activated FVIII mimetic, is a prophylactic treatment for PwHA that has shown effective bleed control in paediatric and adult populations with HA. 12 13 14 15 16 These newer therapeutic options have offered greater personalised treatment regimens by increasing choice and flexibility in prophylactic coverage, though FVIII treatment for breakthrough bleeding events may still be needed. Traditional EHL FVIII products, however, require ongoing twice or thrice weekly infusions, and effective medical management is still complicated by the potential development of inhibitors to FVIII in up to 30% of PwHA. 17 18 Ultralong half-life FVIII products allowing for once-weekly infusions have also become available recently; however, wide access remains limited at this time. 19 20 21

Despite improvements in adherence, convenience, and clinical outcomes observed with therapeutic advances, 22 23 long-standing challenges remain, such as the management of long-term joint health, along with opportunities to further improve clinical outcomes and health-related quality of life (HRQoL) for people with this lifelong condition. 24 To this end, it is particularly important to consider humanistic outcomes along with patient characteristics, real-world treatment patterns and clinical outcomes in order to refine our understanding of how specific treatment attributes may align with the needs of specific subgroups of PwHA. These considerations include, at a minimum, how treatment regimens impact the performance of daily activities, work productivity, and outcomes deemed most important by PwHA. This analysis explored HRQoL, work productivity and other patient-reported outcomes (PROs) among people with severe haemophilia A (PwSHA) in Europe on emicizumab or FVIII prophylaxis.

## Methods

### Data Source and Analysis Population

The “Cost of Haemophilia: a Socio-economic Survey” (CHESS) study is a 12-month retrospective burden-of-illness study of adult men (aged ≥18 years) with congenital HA or haemophilia B of any severity, with or without inhibitors, who were not participating in a clinical trial at the time of data collection or during the preceding 12 months.

Study design, methodology and previous findings have been reported previously. 25 26 27 Briefly, the CHESS study is a retrospective burden of illness study that is carried out periodically with information provided by both people with haemophilia (PwH) and health care providers (HCP). Treating health care providers completed a specifically designed clinical report form (CRF) reporting on demographics, clinical characteristics, treatment patterns and health resource utilisation based on the PwH's medical history, comprising the total population (with CRF data). PwH completed voluntary questionnaire forms reporting on socioeconomic information, as well as their health status, HRQoL, labour market and productivity outcomes, financial and daily life burden information, as well as other PRO measures, comprising the ‘PRO cohort’. Data collection was carried out periodically across five European countries (EU5; France, Germany, Italy, Spain and the United Kingdom) and the United States, with a subsample of participants followed over time and all data anonymised at the source to ensure protection of personal information. 27 Participating HCPs recruit the next eligible 8 to 16 patients they consult with, regardless of consultation reason in order to minimise selection bias. 28 Not all PwHA for whom clinical data were available completed a questionnaire; that is, not all PwSHA in the CRF sample were included in the PRO cohort. All participants provided informed consent and the study protocol was approved by the Research Ethics Sub-committee of the Faculty of Health and Social Care of the University of Chester and received US exemption after review by WIRB-Copernicus Group. The study was carried out in accordance with regional and relevant guidelines.

This analysis used data on CHESS participants from the EU5 with severe HA and no inhibitor diagnosis in the 12 months preceding data collection who were receiving prophylactic treatment (emicizumab or FVIII replacement therapy) from the 2022 (April–November 2022) and 2024 (October 2023–April 2024) study cohorts and specifically the subsample of eligible PwH completing a patient questionnaire. The entire sample, consisting of PwSHA and no inhibitors who had a completed CRF (regardless of patient questionnaire completion), was also evaluated to identify any potential differences in demographic characteristics or clinical outcomes that may have arisen from selective completion of the patient questionnaires by a subset of PwSHA. As individuals participating in clinical trials were not included in this study, no participants were treated with efanesoctocog alfa at the time of data collection.

### Patient Characteristics

Demographic and clinical characteristics of PwSHA, including age, body mass index (BMI), HA treatment regimens and employment status, were collected by the HCPs. HCPs also registered bleeding events, use of on-demand FVIII infusions, joint health and chronic pain for their PwSHA as reported by the PwSHA during routine clinical care visits in the 12 months prior to data collection. Bleeding events included all spontaneous/traumatic joint and non-joint injuries. Annual bleeding rate (ABR) was calculated based on the sum of all bleeding events. Annual spontaneous bleeding rate (ASBR) and annual joint bleeding rate (AJBR) were also calculated as the sum of all spontaneous and joint bleeding events, respectively. On-demand FVIII infusions to treat breakthrough bleeding events were defined as infusions outside the normal prophylaxis treatment schedule that were used to treat an actual or suspected bleeding event. Joint health was captured as Problem Joints 25 (defined as joints with chronic pain and/or limited range of movement due to chronic synovitis or arthropathy, with or without persistent bleeding) and Target Joints. 29 Problem joints were also captured from the point of view of the patient within the patient form, recording the specific symptom affecting them and the relative intensity. Location and magnitude of HA-related chronic pain were classified by the treating HCP using a 4-point scale (0, no pain; 4, severe pain), accounting for functional deficits, interference with activities of daily living, and use of analgesics/narcotics. 30

### Health-Related Quality of Life, Work Productivity and Mental Health

HRQoL data were collected from PwSHA using the validated, historical standard for patient-reported HRQoL EQ-5D-5L instrument, comprising five dimensions evaluating mobility, self-care, usual activities, pain/discomfort and anxiety/depression. 31 Additionally, specific HRQoL-related outcomes were also collected as described below. For this analysis, the United Kingdom's EQ-5D crosswalk was applied using the mapping algorithm developed by Hernandez Alava and colleagues, allowing for observations from all countries to be assessed in aggregation. 32

The impact of SHA on work productivity and activity impairment (WPAI) was captured via the Work Productivity and Activity Impairment Specific Health Problem instrument. 33 Raw scores from the six questions in the tool allow for estimation of an overall impairment, presenteeism, absenteeism and activity impairment percentages. 33 Additional work productivity outcomes included the proportion of each treatment group that reported having missed work in the previous 3 months and the number of workdays missed.

Self-reported mental health is assessed using the GAD-7 34 and the 8-item Patient Health Questionnaire (PHQ-8), 35 assessing symptoms of anxiety and depression over the previous 2 weeks.

The self-reported impact of HA on specific areas of daily life was also captured using four custom-built questions focusing on social activities, physical activities, life opportunities and feelings of frustration with HA's impact on lifestyle based on a 1- to 5-point Likert scale describing the level of compromise in the specific area. 27 Adaptation of treatment dosing and administration based on physical and social activities was also captured in the patient questionnaire, inclusive of additional infusions outside of the habitual treatment schedule that were not related to a specific bleeding event. The requirement for assistance in daily activities was also reported by PwSHA. Full definitions of the outcomes of interest are provided in Supplementary Table S1 .

### Statistical Analysis

All findings were analysed descriptively. For continuous variables, both means (with standard deviations [SDs]) and medians (with ranges) were reported. Between-group differences in means in PRO measures and HRQoL variables were summarised using a two-sample *t* -test with corresponding 95% confidence intervals (CIs), while differences in medians were estimated using bootstrapping to generate 95% CIs. For binary categorical PRO/HRQoL variables, frequencies and proportions were presented, with differences in proportions and 95% CIs estimated using a two-proportion *z* -test. Consistent with the descriptive nature of the study, no formal hypothesis testing was carried out, and no *p* -values are presented. All analyses were performed using STATA 18 (StataCorp LLC, College Station, TX; www.stata.com ).

## Results

### Analysis Population

A total of 350 PwSHA met the eligibility criteria with information provided by their health care providers in the CRF and were included in this analysis. The emicizumab and FVIII prophylaxis groups had similar mean (SD) number of target joints (0.6 [1.0] vs. 0.5 [0.7]) and problem joints (0.8 [1.1] vs. 1.0 [1.3]; Supplementary Table S2 ). All other characteristics of the total population are provided in Supplementary Table S2 .

Approximately one-quarter (27%, *n* = 94) of the total analysis population completed the patient questionnaires and were included in the PRO cohort. Characteristics of the PRO cohort ( Table 1 ) were generally consistent with those of the total analysis population ( Supplementary Table S2 ), but a larger proportion of the PRO cohort was receiving emicizumab (68% and 55%, respectively). The PRO cohort also appeared to be slightly older, with mean (SD) ages of 38.8 (14.3) and 39.9 (16.1) for the emicizumab and FVIII groups, respectively, in the PRO cohort, and 34.0 (13.2) and 36.1 (12.9) in the total population. Additionally, albeit generally comparable, slightly less favourable clinical characteristics were noted in the PRO cohort, such as a mean (SD) ABR of 1.6 (1.4) and 1.4 (1.1) in the emicizumab and FVIII groups, respectively, compared with 1.3 (1.3) and 1.2 (1.0) in the total population ( Table 1 and Supplementary Table S2 ).

**Table 1:** Demographic and clinical characteristics of people with severe haemophilia A in the patient-reported outcome cohort ( *n* = 94)

	Emicizumab ( *n* = 64)	FVIII prophylaxis ( *n* = 30)
Age, years, mean (SD)	38.8 (14.3)	39.9 (16.1)
BMI, kg/m ^2^ , mean (SD)	24.2 (3.8)	25.1 (2.9)
Country, *n* (%)		
Italy	26 (40.6)	15 (50.0)
Spain	7 (10.9)	7 (23.3)
United Kingdom	11 (17.2)	2 (6.7)
France	14 (21.9)	0
Germany	6 (9.4)	6 (20.0)
Employment, *n* (%)		
Employed	49 (76.6)	23 (76.7)
Student	4 (6.2)	2 (6.7)
Not employed	8 (12.5)	5 (16.7)
Physically unable to work due to HA	1 (1.6)	0
Unknown	2 (3.1)	0
Education, *n* (%)		
None or primary schooling only	0 (0.0)	1 (3.3)
Secondary, Vocational, High School	39 (60.9)	16 (53.3)
University	23 (35.9)	12 (40.0)
Other or do not know	2 (3.1)	1 (3.3)
Ethnic origin, *n* (%) a		
White/Caucasian	47 (94.0)	29 (96.7)
Black/Afro-Caribbean	2 (4.0)	0 (0.0)
Middle Eastern	0 (0.0)	1 (3.3)
Asian–Indian subcontinent	1 (2.0)	0 (0.0)
FVIII treatment, *n* (%)		
SHL/Plasma-derived	–	13 (43.3)
EHL	–	17 (56.7)
Previous treatment strategy, *n* (%)		
Prophylaxis b	40 (62.5)	23 (76.7)
High-dose FVIII prophylaxis c	12 (30.0)	16 (69.6)
Intermediate-dose FVIII prophylaxis d	6 (15.0)	5 (21.7)
Other e	22 (55.0)	2 (8.7)
On-demand	24 (37.5)	7 (23.3)
Treatment class or strategy switch in 12 months prior, *n* (%)	19 (29.7)	6 (20.0)
Received ≥1 FVIII infusion for breakthrough bleeding events, *n* (%) Mean (SD) number of infusions	22 (34.4) 0.5 (1.2)	10 (55.6) 2.7 (3.2)
Target joints, number, mean (SD)	0.6 (1.0)	0.4 (0.7)
Proportion with 1+ problem joints, *n* (%) Problem joints, number, mean (SD)	29 (45.3%) 0.8 (1.1)	17 (56.7%) 1.0 (1.3)
PwSHA report of 1+ problem joint, *n* (%) PwSHA-reported Problem Joint symptom, *n* (%) f Chronic pain Chronic synovitis Range of movement restriction Recurrent bleeding Haemophilic arthropathy	39 (60.9%) 30 (46.9%) 8 (12.5%) 17 (26.6%) 4 (6.2%) 3 (4.7%)	19 (63.3%) 16 (53.3%) 2 (6.7%) 11 (36.37%) 3 (10.0%) 1 (3.3%)
Chronic pain, *n* (%)		
None	24 (37.5)	9 (30.0)
Mild	26 (40.6)	10 (33.3)
Moderate	13 (20.3)	11 (36.7)
Severe	1 (1.6)	0
ABR, mean (SD)	1.6 (1.4)	1.4 (1.1)
AJBR, mean (SD)	0.7 (1.0)	0.8 (1.0)
ASBR, mean (SD)	1.0 (1.2)	0.7 (0.8)
Treated annualised ABR (≥3 months on treatment), *n* , mean (SD)	*n* = 60, 0.6 (1.2)	*n* = 18, 0.8 (0.9)
Number of infusions to resolve bleeding episode, mean (SD)	*n* = 60, 0.6 (1.2)	*n* = 18, 2.7 (3.2)
Annualised ABR on previous treatment, *n* , mean (SD)	*n* = 56, 2.3 (2.5)	–
ABR difference (pre- vs. posttreatment), *n* , mean (SD)	*n* = 53, −0.6 (1.5)	–

Abbreviations: ABR, annual bleeding rate; AJBR, annual joint bleeding rate; ASBR, annual spontaneous bleeding rate; BMI, body mass index; EHL, extended half-life; FVIII, factor VIII; HA, haemophilia A; PwSHA, person with severe HA; SD, standard deviation; SHL, standard half-life.

All variables reported by HCPs unless otherwise specified.

a Ethnic origin data were not available for 14 patients in the emicizumab group.

b May include any product type (standard half-life FVIII, extended half-life FVIII, plasma-derived FVIII or emicizumab).

c High-dose prophylaxis is defined as >4,000 IU/kg/year. 8

d Intermediate-dose prophylaxis is defined as 1,500 to 4,000 IU/kg/year; 8

e Comprises low-dose prophylaxis (<1,500 IU/kg/year; *n* = 4, 6.4%), 8 PwSHA previously on a different frequency of emicizumab ( *n* = 7, 11.1%) and PwSHA where the information was missing ( *n* = 13, 20.6%).

f Proportion of people with haemophilia A reporting the specific symptom in at least one of their reported problem joints.

### Characteristics of the Patient-Reported Outcome Cohort

Of the PRO cohort, 68% ( *n* = 64) were receiving emicizumab and 32% ( *n* = 30) were receiving FVIII prophylaxis. Within the FVIII prophylaxis group, 57% ( *n* = 17) were using EHL products. Overall, the greatest proportion of PwSHA were in Italy (43%, *n* = 41), and the rest of the sample was generally evenly distributed across the other four countries, ranging from 13% ( *n* = 12) in Germany to 15% ( *n* = 14) in Spain ( Table 1 ). Greater proportions of the emicizumab group were in France and the United Kingdom, and more of the FVIII group were in Italy and Spain. Mean (SD) ages of PwSHA were 38.8 (14.3) and 39.9 (16.1) years for the emicizumab and FVIII prophylaxis groups, respectively. Mean BMI was 24.2 (3.8) and 25.1 (2.9) kg/m ^2^ , with 59% and 57% of the emicizumab and FVIII treatment groups in the healthy weight range, respectively. In both treatment groups, 77% of PwSHA were employed, with 36% ( *n* = 23) and 40% ( *n* = 12) of the emicizumab and FVIII prophylaxis groups educated at the university level ( Table 1 ).

The majority of PwSHA in both the emicizumab and FVIII prophylaxis groups reported some level of chronic pain (63% vs. 70%, respectively). Chronic pain was also the most commonly reported symptom among PwSHA who reported suffering from problem joints in both the emicizumab (61%, *n* = 39) and FVIII prophylaxis groups (63%, *n* = 19; Table 1 ). Bleeding outcomes were largely equivalent between the treatment groups in terms of ABR, AJBR, ASBR and treated bleeding events. On-demand infusions to treat breakthrough bleeding events, however, were reported for 34% ( *n* = 22) and 56% ( *n* = 10) of PwSHA receiving emicizumab or FVIII prophylaxis who had available information (the number of infusions to treat breakthrough bleeding events was only available for 82 patients PwSHA in this sample; 18 in the FVIII prophylaxis group and 64 in the emicizumab group). Mean (SD) number of on-demand infusions to achieve bleed resolution was 0.6 (1.2) and 2.7 (3.2) for the emicizumab and FVIII prophylaxis groups. A greater proportion of PwSHA in the emicizumab group were previously on an on-demand treatment regimen (38% vs. 23%), with 30% versus 20% switching their treatment regimens in the previous 12 months. Most patients with a history of prophylaxis treatment and available information had received high-dose prophylaxis regimens ( Table 1 ). Compared to previous treatments, PwSHA taking emicizumab experienced a decrease of 0.6 bleeds per year ( Table 1 ).

### Mental Health

**Fig. 1:**
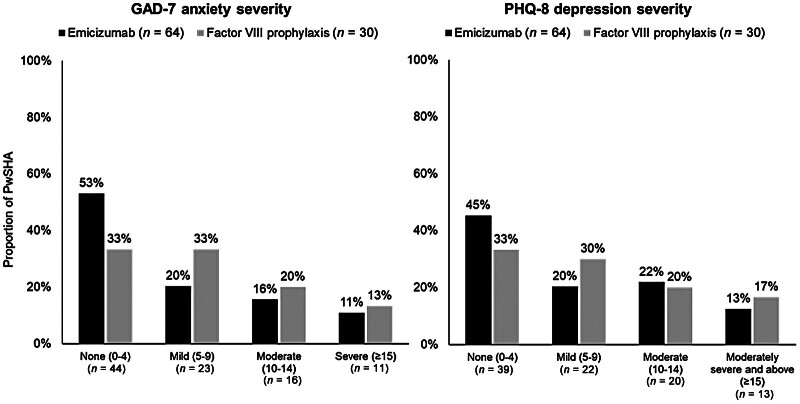
Proportions of PwSHA reporting anxiety and depression. Proportions may not sum to 100% due to rounding. Maximum scores are 21 for the GAD-7 and 24 for the PHQ-8; both characterise ‘moderate’ or worse anxiety or depression as scores ≥10. 34 35 GAD-7, 7-item General Anxiety Disorder questionnaire; PHQ-8, 8-item Patient Health Questionnaire; PwSHA, people with severe haemophilia A.

PwSHA in the emicizumab group reported less impact of SHA on their mental health than those receiving FVIII prophylaxis ( Fig. 1 and Table 2 ). Specifically, GAD-7 anxiety scores were numerically lower in the emicizumab group compared with the FVIII prophylaxis group (mean [SD], 6.0 [5.8] vs. 7.3 [5.2]; Table 2 ). Half (53%) of the emicizumab group reported no anxiety compared with 33% of the FVIII prophylaxis group, and a smaller proportion self-reported their anxiety levels as mild or worse (47%, *n* = 30) compared with 67% ( *n* = 20) of the FVIII prophylaxis group. This observation remained when median scores were analysed, showing a median score for the emicizumab group of 4.0 (range, 0–18) and a median score for the FVIII prophylaxis group of 6.0 (range 0–19), where mild scores for the GAD-7 range from 5 to 9, moderate scores from 10 to 14 and severe indicated by a score of ≥15 ( Table 2 ).

**Table 2:** Health-related quality of life, work productivity and activity impairment and mental health outcomes among people with severe haemophilia A

	Emicizumab ( *n* = 64)	FVIII prophylaxis ( *n* = 30)	Delta (95% CI) c
EQ-5D-5L index score Mean (SD) Median (range)	0.71 (0.25) 0.72 (−0.1–1.0)	0.69 (0.29) 0.70 (−0.3–1.0)	−0.02 (−0.14–0.09) −0.02 (−0.15–0.12)
Impact on work, previous 3 months Missed ≥1 workday, *n* (%)	10 (15.6)	12 (40.0)	24.4 (4.7–44.0)
Number of days missed Mean (SD) Median (range)	*n* = 10 3.7 (2.1) 3.0 (2–8)	*n* = 12 4.2 (2.4) 4.5 (1–8)	0.6 (−1.5–2.6) 1.5 (−1.2–4.2)
Did not miss days but experienced issues in carrying out tasks, *n* (%)	22 (34.4)	6 (20.0)	−14.4 (−32.8–4.1)
WPAI a			
Overall work productivity loss b Mean (SD) Median (range)	*n* = 51 31.3 (30.7) 26.2 (0–92)	*n* = 18 50.1 (24.3) 56.1 (0–83)	18.8 (2.8–34.8) 29.9 (9.6–50.3)
Absenteeism b Mean (SD) Median (range)	*n* = 51 6.7 (12.5) 0.0 (0–60)	*n* = 18 16.5 (20.5) 8.3 (0–67)	9.9 (1.7–18.0) 8.3 (−2.4–19.1)
Presenteeism b Mean (SD) Median (range)	*n* = 51 28.8 (28.5) 20.0 (0–90)	*n* = 18 41.1 (21.1) 45.0 (0–70)	12.3 (−2.4–27.0) 25.0 (6.0–44.0)
Activity impairment Mean (SD) Median (range)	39.8 (30.5) 40.0 (0–100)	48.7 (26.0) 55.0 (0–100)	8.8 (−4.0–21.6) 15.0 (−6.6–36.6)
GAD-7 score Mean (SD) Median (range)	6.0 (5.8) 4.0 (0–19)	7.3 (5.2) 6.0 (0–18)	1.3 (−1.2–3.8) 2.0 (−2.1–6.1)
GAD-7 anxiety severity category, *n* (%) d None (0–4) Mild (5–9) Moderate (10–14) Severe (≥15)	34 (53.1) 13 (20.3) 10 (15.6) 7 (10.9)	10 (33.3) 10 (33.3) 6 (20.0) 4 (13.3)	–
PHQ-8 score Mean (SD) Median (range)	6.8 (6.0) 5.0 (0–22)	7.8 (5.7) 7.0 (0–21)	1.1 (−1.6–3.7) 2.0 (−2.0–6.0)
PHQ-8 depression severity category, *n* (%) d None (0–4) Mild (5–9) Moderate (10–14) Moderately severe and above (≥15)	29 (45.3) 13 (20.3) 14 (21.9) 8 (12.5)	10 (33.3) 9 (30.0) 6 (20.0) 5 (16.7)	–

Abbreviations: CI, confidence interval; FVIII, factor VIII; GAD-7, 7-item General Anxiety Disorder questionnaire; HA, haemophilia A; PHQ-8, 8-item Patient Health Questionnaire; SD, standard d

eviation; WPAI, work productivity and activity impairment.

a Delta values were calculated as the difference between group means, medians and cohort proportions (FVIII prophylaxis cohort minus emicizumab cohort); small discrepancies (≤0.1) may be observed due to rounding of individual values. Delta values and corresponding 95% CIs for means, medians, and proportions were calculated using *t* -test, bootstrapping, and *z* -test, respectively.

b WPAI scores should be interpreted as the percentage of impairment in each category.

c Emicizumab group, *n* = 51; FVIII group, *n* = 18.

d Delta values and corresponding CIs were not computed for these variables due to the small sample size within each subgroup and the absence of a strong clinical rationale for further grouping.

A similar trend was observed for PHQ-8 depression scores, but with a slightly higher level of burden for both groups and lower scores for the emicizumab group compared with the FVIII prophylaxis group (mean [SD], 6.8 [6.1] vs. 7.8 [5.7]; Fig. 1 and Table 2 ). This observation remained when median scores were analysed, showing a median score in the emicizumab group of 5.0 (range, 0–22) and a median score in the FVIII prophylaxis group of 6.0 (range 0–21; Table 2 ). As with the GAD-7, mild scores for the PHQ-8 range from 5 to 9, moderate scores from 10 to 14 and moderately severe and above are indicated by a score of ≥15. A larger proportion of PwSHA in the FVIII prophylaxis group (67%) reported some level of depression (PHQ-8 score >4) compared with 55% of the emicizumab group ( Table 2 ).

### Health-Related Quality of Life and Work Productivity

**Fig. 2:**
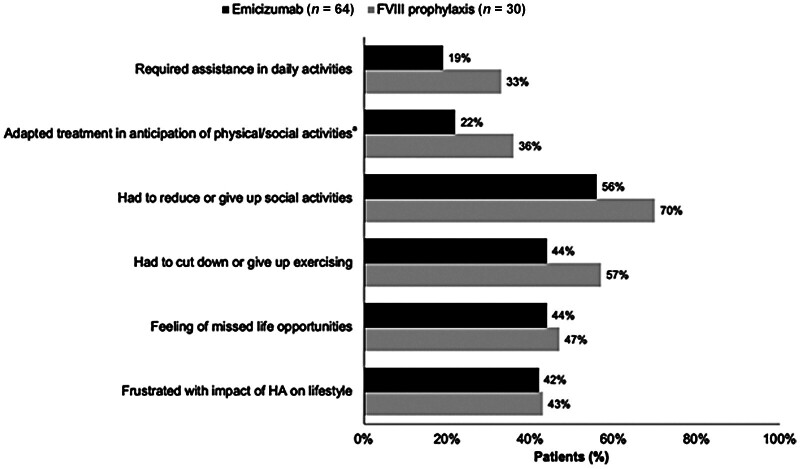
Assistance requirement, treatment adaptation and areas of compromise among PwSHA. ^a^ Missing responses in both the emicizumab and FVIII prophylaxis groups: *n* = 13/64 (22%) for emicizumab, *n* = 9/25 (36%) for FVIII prophylaxis. FVIII, factor VIII; PwSHA, people with severe haemophilia A.

Overall, HRQoL showed comparable EQ-5D-5L scores between the treatment groups, with a marginally higher mean score in the emicizumab group (0.71 vs. 0.69) compared with the FVIII prophylaxis group ( Table 2 ). The emicizumab group also reported lower activity impairment, on average, when compared with the FVIII prophylaxis group (39.8 vs. 48.7). A similar trend was observed in treatment adaptation to physical and social activities, with a smaller proportion of the emicizumab group looking to adapt their treatment (22%, *n* = 13) compared with the FVIII prophylaxis group (36%, *n* = 9). A smaller proportion of the emicizumab group also required assistance with daily activities compared with the FVIII prophylaxis group (19% vs. 33%; Fig. 2 and Table 2 ).

**Fig. 3:**
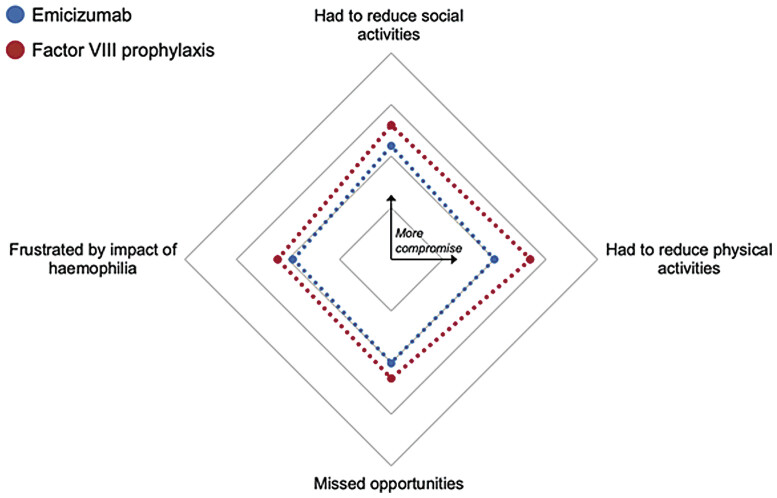
Haemophilia-related compromises reported by PwSHA. Points further from the centre indicate greater burden/compromise. PwSHA, people with severe haemophilia A.

Substantial proportions of both treatment groups reported believing that haemophilia had caused some level of compromise in their daily lives, however, this was a larger proportion of the FVIII prophylaxis group (53%, *n* = 16) than the emicizumab group (44%, *n* = 28; Fig. 2 ). Approximately 45% of each group reported feeling frustrated with the impact of haemophilia on their lifestyle and missing out on opportunities due to their haemophilia ( Fig. 2 ). The FVIII prophylaxis group, however, reported feeling more impacted in the areas of social activities (70% vs. 56%) and physical activity (57% vs. 44%) than the emicizumab group, and a greater proportion had to give up exercise (57%) than in the emicizumab group (44%; Fig. 2 ). Across all areas of compromise, however, the emicizumab cohort reported on average, lower compromise ( Fig. 3 ).

The impact of haemophilia on employed PwSHA was lower for the emicizumab group, with overall work impairment reported to be 31% versus 50% ( Table 2 ). Specifically, lower rates of both mean (SD) absenteeism (6.7 [12.5] vs. 16.6 [20.5]) and presenteeism (28.8 [28.5] vs. 41.1 [21.1]) were reported with emicizumab compared with the FVIII prophylaxis group. A smaller proportion of the emicizumab group also reported having missed work in the preceding 3 months (16% vs. 40%), with a mean (SD) of 3.7 (2.1) vs. 4.2 (2.4) missed workdays during that time ( Table 2 ). Among those not reporting any missed work, however, substantial proportions of PwSHA reported problems carrying out tasks at work due to their haemophilia, which appeared higher in the emicizumab group (34% and 20% of the emicizumab and FVIII prophylaxis groups, respectively; Table 2 ).

## Discussion

This analysis of a cohort of PwSHA receiving prophylaxis in the CHESS III–IV study showed a substantial burden of haemophilia on the mental health, work productivity and HRQoL of PwSHA in Europe. Most PwSHA reported at least a mild degree of anxiety and depression, which was numerically less of a burden for those on an emicizumab compared to FVIII prophylaxis regimen. Among employed PwSHA, overall work productivity impairment was notable and lower among those receiving emicizumab. Only 16% of PwSHA receiving emicizumab had missed at least one day of work in the previous 3 months, with a mean of 3.7 days missed, compared with 40% of those receiving FVIII prophylaxis (mean, 4.2 days missed). Most PwSHA reported making compromises to their social lives, ability to exercise and other life opportunities due to their SHA, which was again reported more among those on an FVIII prophylaxis regimen than those taking emicizumab. These trends were observed in the context of similar clinical outcomes, including ABR, joint health and chronic pain between the treatment groups, despite evidence suggesting PwSHA in the emicizumab group may have had more morbidity in their medical and treatment history.

Overall, these data demonstrate the importance of treatment burden and good haemostatic control on HRQoL, supporting the role of emicizumab as part of the standard of care in clinical practice. 36 These findings are also consistent with previous reports of HRQoL improvements observed with emicizumab among PwHA without inhibitors in the HAVEN clinical trial program, 37 38 including improvements in pain and pain-related HRQoL within 13 weeks of starting treatment that were maintained through 78 weeks. 39

In fact, the PROs suggested that emicizumab may provide distinct advantages in non-clinical aspects of the lives of PwSHA. PwSHA receiving an emicizumab regimen also reported less need for caregiver support compared with the FVIII prophylaxis group. PwSHA in the emicizumab group appeared to have a more complex treatment history than their counterparts in the FVIII prophylaxis group, with 38% versus 23% previously treated with on-demand regimens, and 30% versus 20% having undergone a treatment switch in the 12 months prior, consistent with a previous analysis of claims data, hypothesising channelling bias in emicizumab patients. 40 This may suggest added benefits of emicizumab for PwSHA who have or have had treatment-related challenges (e.g., venous access, needle phobia, adherence issues, unfavourable patient-specific pharmacokinetics) or in balancing their condition with personal and professional engagements. In these instances, emicizumab may provide an efficacious therapeutic option that reduces treatment burden while providing appropriate protection from bleeding events and supporting better mental health, work productivity and HRQoL.

Employed PwSHA in both treatment groups reported an impact of SHA on work productivity. However, consistent with previous findings, 37 PwSHA taking emicizumab reported greater work productivity with fewer missed workdays and lower rates of both presenteeism and absenteeism compared with the FVIII prophylaxis group. In-line with this finding, PwSHA on emicizumab reported less impairment of daily life activities than their counterparts taking FVIII factor prophylaxis. However, approximately half of each treatment group reported missing out on life opportunities due to their haemophilia and feeling frustrated with the impact of haemophilia on their daily lives. More of the FVIII prophylaxis group, however, reported adapting their treatment to account for physical and social activities, and reducing their social and physical aspects of life due to their haemophilia, compared with those taking emicizumab.

Lower levels of anxiety and depression were observed in the emicizumab group than in the FVIII prophylaxis group, potentially reflecting the positive effect of a lower treatment burden in the context of appropriate bleed control. Previous research has linked frequent treatment administrations with reduced quality of life and treatment burden to potential mental health impact on PwH, suggesting that a reduced treatment burden may help to improve emotional well-being. 24 26 41 42 In contrast, Pedra and colleagues reported how more frequent FVIII treatment administration may be beneficial in PwSHA who have adverse clinical situations, particularly those with severe chronic pain, highlighting the importance of tailoring treatment to the specific clinical situation of the PwSHA to achieve optimal outcomes. 43 Although mental health and HRQoL are critical components of the haemophilia care pathway, and are not often captured quantitatively in routine practice, these data advocate for the use of these simple tools as part of routine haemophilia management provided by the multidisciplinary care team. 28 Assessments at least once every 12 to 24 months can prompt discussions between the patient and their care team around aspects of management, including treatment burden and treatment anxiety. All of these should be taken into consideration along with lifestyle, social support, patient preferences and clinical considerations in a shared decision-making process between PwSHA and their care team. Such a patient-centred and holistic approach to haemophilia treatment can contribute to improving clinical, quality of life and emotional well-being outcomes. Future research should continue to explore the impact of different prophylactic treatments on both clinical and humanistic outcomes and the impact of such assessments in changing these outcomes.

These findings should be considered in the context of certain limitations. This was an analysis of secondary data that were not collected for the specific and sole purpose of this analysis. The CHESS study was not designed a priori to detect statistically significant differences between treatment groups. For this reason, we refrained from performing any statistical tests and have only reported numerical differences. As this was a secondary analysis of a study with no formal hypotheses or prespecified power calculations, statistical power may be limited. Accordingly, and in-line with the descriptive and exploratory nature of this analysis, no formal hypothesis testing was conducted. Despite these limitations, these findings offer valuable insight into the potential magnitude of differences between the two treatment groups and may provide a foundation for hypothesis generation in future studies. Further, patient and clinician participation in the CHESS studies was voluntary, and a degree of selection bias was possible. Specifically, PwSHA with a more complicated clinical situation may be more likely to visit the treatment centre, therefore increasing the likelihood of being offered and completing the questionnaire, potentially contributing to selection bias. It should also be noted that PwSHA taking emicizumab comprised a larger proportion of the PRO cohort (68%) compared with the CRF sample (55%), which may have influenced the direction of findings. A degree of recall bias was also possible, as well as potential data entry errors or incomplete medical records, which may lead to underestimation of clinical outcomes. The study's descriptive design does not account for potential confounding factors due to differences in baseline characteristics between the treatment groups. PwSHA taking emicizumab may generally have a more complicated treatment history, which could influence the outcomes. Ideally, matching or weighting methods should be used in future work to balance groups by their baseline characteristics for a robust comparison. However, these methods were not adopted due to the small sample size. As a result, while differences in ABR were observed between the groups, it was not possible to infer whether these differences were driven by variations in activity levels, joint health, or prior treatment efficacy. Additionally, variation in treatment type (standard half-life vs. EHL) within the FVIII prophylaxis cohort may have introduced further confounding; however, subgroup analyses to explore this were not feasible due to the limited sample size. Furthermore, the primary focus of this analysis was to assess WPAI, EQ-5D, anxiety and depression outcomes rather than to draw conclusions on the drivers of bleeding rates. Finally, owing to the relatively small size of the analysis sample, the generalisability of these results may be limited, and the results may not be representative of the wider population of PwSHA. Therefore, caution is warranted in the interpretation of results. Despite these limitations, this analysis provides important preliminary evidence in the absence of extensive literature on this topic, highlighting the need for further investigation and serving as a basis for future research in this area. Future research is warranted to further explore specific aspects of treatment on PwSHA, including how patients start and sustain prophylaxis regimens in the context of emicizumab availability, and how the long-term effects of different prophylactic regimens impact both clinical and PROs.

## Conclusion

PwSHA receiving emicizumab prophylaxis regimens appeared to have more favourable mental health, work productivity and HRQoL-related outcomes than those receiving FVIII prophylaxis. These findings were observed in the context of comparable clinical characteristics between emicizumab and FVIII prophylaxis despite evidence of a more complex treatment history for the emicizumab group. Larger studies, accounting for potential confounding, should compare HRQoL longitudinally across novel treatments.

## References

[1] Data and Demographics Committee of the World Federation of Hemophilia Iorio A Stonebraker J S Chambost H Establishing the prevalence and prevalence at birth of hemophilia in males: A meta-analytic approach using national registries Ann Intern Med 2019 171 08 540 546 31499529 10.7326/M19-1208

[2] Soucie J M Miller C H Dupervil B Le B Buckner T W Occurrence rates of haemophilia among males in the United States based on surveillance conducted in specialized haemophilia treatment centres Haemophilia 2020 26 03 487 493 32329553 10.1111/hae.13998 PMC8117262

[3] Berntorp E Future of haemophilia outcome assessment: registries are key to optimized treatment J Intern Med 2016 279 06 498 501 27199237 10.1111/joim.12504

[4] Chowdary P Nissen F Burke T The humanistic and economic burden of problem joints for children and adults with moderate or severe haemophilia A: Analysis of the CHESS population studies Haemophilia 2023 29 03 753 760 36897517 10.1111/hae.14766

[5] O'Hara J Noone D Watt M Relationship between bleeding episodes, health-related quality of life and direct costs in adults with severe haemophilia A: Secondary analyses from the CHESS study Haemophilia 2022 28 05 e117 e120 35773741 10.1111/hae.14616 PMC9545054

[6] Hemophilia Treatment Centers Network Soucie J M Grosse S D Siddiqi A E The effects of joint disease, inhibitors and other complications on health-related quality of life among males with severe haemophilia A in the United States Haemophilia 2017 23 04 e287 e293 28574229 10.1111/hae.13275 PMC5533283

[7] O'Hara J Sima C S Frimpter J Paliargues F Chu P Presch I Long-term outcomes from prophylactic or episodic treatment of haemophilia A: A systematic review Haemophilia 2018 24 05 e301 e311 30004613 10.1111/hae.13546

[8] WFH Guidelines for the Management of Hemophilia panelists and co-authors Srivastava A Santagostino E Dougall A WFH Guidelines for the Management of Hemophilia, 3rd edition Haemophilia 2020 26 06 1 158 32744769 10.1111/hae.14046

[9] Manco-Johnson M J Kempton C L Reding M T Randomized, controlled, parallel-group trial of routine prophylaxis vs. on-demand treatment with sucrose-formulated recombinant factor VIII in adults with severe hemophilia A (SPINART) J Thromb Haemost 2013 11 06 1119 1127 23528101 10.1111/jth.12202

[10] Oladapo A O Epstein J D Williams E Ito D Gringeri A Valentino L A Health-related quality of life assessment in haemophilia patients on prophylaxis therapy: a systematic review of results from prospective clinical trials Haemophilia 2015 21 05 e344 e358 26390060 10.1111/hae.12759

[11] Thornburg C D Duncan N A Treatment adherence in hemophilia Patient Prefer Adherence 2017 11 1677 1686 29033555 10.2147/PPA.S139851 PMC5630068

[12] Barg A A Budnik I Avishai E Emicizumab prophylaxis: Prospective longitudinal real-world follow-up and monitoring Haemophilia 2021 27 03 383 391 33892524 10.1111/hae.14318

[13] Knight T Callaghan M U The role of emicizumab, a bispecific factor IXa- and factor X-directed antibody, for the prevention of bleeding episodes in patients with hemophilia A Ther Adv Hematol 2018 9 10 319 334 30344994 10.1177/2040620718799997 PMC6187429

[14] Mancuso M E Santagostino E Outcome of clinical trials with new extended half-life FVIII/IX concentrates J Clin Med 2017 6 04 39 28350322 10.3390/jcm6040039 PMC5406771

[15] PedNet Investigators van der Zwet K de Kovel M Motwani J Bleeding control improves after switching to emicizumab: Real-world experience of 177 children in the PedNet registry Haemophilia 2024 30 03 685 692 38578720 10.1111/hae.15015

[16] UK Haemophilia Centre Doctors' Organisation (UKHCDO) Wall C Xiang H Palmer B Emicizumab prophylaxis in haemophilia A with inhibitors: Three years follow-up from the UK Haemophilia Centre Doctors' Organisation (UKHCDO) Haemophilia 2023 29 03 743 752 36811304 10.1111/hae.14762

[17] Iorio A Halimeh S Holzhauer S Rate of inhibitor development in previously untreated hemophilia A patients treated with plasma-derived or recombinant factor VIII concentrates: a systematic review J Thromb Haemost 2010 8 06 1256 1265 20345722 10.1111/j.1538-7836.2010.03823.x

[18] Young G Pipe S W Kenet G Emicizumab is well tolerated and effective in people with congenital hemophilia A regardless of age, severity of disease, or inhibitor status: a scoping review Res Pract Thromb Haemost 2024 8 04 102415 38812987 10.1016/j.rpth.2024.102415 PMC11135026

[19] XTEND-1 Trial Group von Drygalski A Chowdary P Kulkarni R Efanesoctocog alfa prophylaxis for patients with severe hemophilia A N Engl J Med 2023 388 04 310 318 36720133 10.1056/NEJMoa2209226

[20] Sobi European Commission grants Sobi® Marketing Authorisation for ALTUVOCT™ for treatment of haemophilia A 2024

[21] Sanofi FDA approves once-weekly ALTUVIIIO™, a new class of factor VIII therapy for hemophilia A that offers significant bleed protection 2023

[22] rAHF-PFM Study Group Collins P W Blanchette V S Fischer K Break-through bleeding in relation to predicted factor VIII levels in patients receiving prophylactic treatment for severe hemophilia A J Thromb Haemost 2009 7 03 413 420 19143924 10.1111/j.1538-7836.2008.03270.x

[23] Warren B B Blades T Smith N L Wang M Manco-Johnson M J Breakthrough bleeding in hemophilia A patients on prophylaxis Blood 2016 128 22 2581 2581

[24] Wells J R Gater A Marshall C Tritton T Vashi P Kessabi S Exploring the impact of infusion frequency in hemophilia A: Exit interviews with patients participating in BAY 94-9027 Extension Studies (PROTECT VIII) Patient 2019 12 06 611 619 31313270 10.1007/s40271-019-00374-x PMC6884429

[25] Burke T Rodriguez-Santana I Chowdary P Humanistic burden of problem joints for children and adults with haemophilia Haemophilia 2023 29 02 608 618 36574369 10.1111/hae.14731

[26] Ferri Grazzi E Blenkiron T Aragon M J PCR192 treatment burden and its relationship with health-related quality of life, work productivity and activity impairment in adults with severe non-inhibitor hemophilia A in the United States: Data analysis from the CHESS US+ Study Value Health 2022 25 12 S427

[27] O'Hara J Hughes D Camp C Burke T Carroll L Diego D G The cost of severe haemophilia in Europe: the CHESS study Orphanet J Rare Dis 2017 12 01 106 28569181 10.1186/s13023-017-0660-y PMC5452407

[28] Ferri Grazzi E Blenkiron T Hawes C Anxiety and depression among adults with haemophilia A: Patient and physician reported symptoms from the real-world European CHESS II study Haemophilia 2024 30 03 743 751 38507035 10.1111/hae.14989

[29] Subcommittee on Factor VIII, Factor IX and Rare Coagulation Disorders of the Scientific and Standardization Committee of the International Society on Thrombosis and Hemostasis Blanchette V S Key N S Ljung L R Manco-Johnson M J van den Berg H M Srivastava A Definitions in hemophilia: communication from the SSC of the ISTH J Thromb Haemost 2014 12 11 1935 1939 25059285 10.1111/jth.12672

[30] Gilbert M S Prophylaxis: musculoskeletal evaluation Semin Hematol 1993 30(3, Suppl 2):3 6 8367740

[31] Herdman M Gudex C Lloyd A Development and preliminary testing of the new five-level version of EQ-5D (EQ-5D-5L) Qual Life Res 2011 20 10 1727 1736 21479777 10.1007/s11136-011-9903-x PMC3220807

[32] Hernández Alava M Wailoo A J Ara R Tails from the peak district: adjusted limited dependent variable mixture models of EQ-5D questionnaire health state utility values Value Health 2012 15 03 550 561 22583466 10.1016/j.jval.2011.12.014

[33] Reilly M C Zbrozek A S Dukes E M The validity and reproducibility of a work productivity and activity impairment instrument PharmacoEconomics 1993 4 05 353 365 10146874 10.2165/00019053-199304050-00006

[34] Spitzer R L Kroenke K Williams J B Löwe B A brief measure for assessing generalized anxiety disorder: the GAD-7 Arch Intern Med 2006 166 10 1092 1097 16717171 10.1001/archinte.166.10.1092

[35] Kroenke K Strine T W Spitzer R L Williams J B Berry J T Mokdad A H The PHQ-8 as a measure of current depression in the general population J Affect Disord 2009 114(1-3):163 173 18752852 10.1016/j.jad.2008.06.026

[36] Rezende S M Neumann I Angchaisuksiri P International Society on Thrombosis and Haemostasis clinical practice guideline for treatment of congenital hemophilia A and B based on the Grading of Recommendations Assessment, Development, and Evaluation methodology J Thromb Haemost 2024 22 09 2629 2652 39043543 10.1016/j.jtha.2024.05.026

[37] Skinner M W Négrier C Paz-Priel I The effect of emicizumab prophylaxis on long-term, self-reported physical health in persons with haemophilia A without factor VIII inhibitors in the HAVEN 3 and HAVEN 4 studies Haemophilia 2021 27 05 854 865 34171159 10.1111/hae.14363 PMC8518882

[38] Mahlangu J Oldenburg J Paz-Priel I Emicizumab prophylaxis in patients who have hemophilia A without inhibitors N Engl J Med 2018 379 09 811 822 30157389 10.1056/NEJMoa1803550

[39] Hermans C Skinner M W Gentile B Pain-related quality of life outcomes in people with haemophilia A receiving emicizumab: A post hoc analysis of the HAVEN 1, 3 and 4 and STASEY studies Haemophilia 2025 31 01 87 98 39692401 10.1111/hae.15134 PMC11780222

[40] Mahajerin A Faghmous I Kuebler P Channeling effects in the prescription of new therapies: The case of emicizumab for hemophilia A J Comp Eff Res 2022 11 10 717 728 35535702 10.2217/cer-2021-0278

[41] Al-Huniti A Reyes Hernandez M Ten Eyck P Staber J M Mental health disorders in haemophilia: Systematic literature review and meta-analysis Haemophilia 2020 26 03 431 442 32307801 10.1111/hae.13960 PMC8475067

[42] Rodriguez-Santana I Bartelt-Hofer J Kragh N Aragon M J RWD119 exploring the relationship between prophylactic infusion frequency and patient utility for people with haemophilia in Europe Value Health 2022 25 12 S472

[43] Pedra G Christoffersen P Khair K The impact of factor infusion frequency on health-related quality of life in people with haemophilia J Haemoph Pract 2020 7 01 102 109

