# Supplementary material for: Mental Health, Work Productivity, and Quality of Life in People with Severe Haemophilia A Receiving Prophylaxis: Findings from the CHESS Data Platform
Source: TH Open. 2025 Aug 4;9:a26586151. doi: 10.1055/a-2658-6151 (PMC12509260; doi:10.1055/a-2658-6151)
Supplement: Supplementary file 1 — Supplementary Material [file 10-1055-a-2658-6151_26757483.pdf]

Supplementary Table S1. Variable definitions and stratifications

Variable	Definitions
ABR	HCP-reported; Annual bleed rate (ABR) is the sum of minor bleeding events (characterised by mild pain, minimal swelling, minimal restriction of motion and spontaneous resolution or response within 24 hours of treatment) and major bleeding events (characterised by pain, effusion, swelling, restricted range of motion and failure to respond to treatment within 24 hours) within the past 12 months.
AJBR	HCP-reported; Annual joint bleed rate (AJBR) is measured as the sum of joint bleeding events in the preceding 12 months.
ASBR	HCP-reported; Annual spontaneous bleed rate (ASBR) is measured as the sum of spontaneous bleeding events in the preceding 12 months.
Annualised bleed rate	HCP-reported; for PwH on current treatment for 12 months or longer, ABR reflects the total number of bleeds observed during a full 12-month period. Where treatment duration < 12 months, annualised bleeding rate was calculated as the number of reported bleeding events while on current treatment divided by the number of months in the reporting time window (3-11 months) and multiplied by 12, when the PwH had been on the treatment for at least 3 months.
Treatment strategy	HCP-reported; Prophylaxis: Treatment for the prevention of bleeding event either with FVIII concentrates or with emicizumab<br>On-demand: acute treatment of bleeding events that have already occurred with FVIII concentrates

TH Open | DOI 10.1055/a-2658-6151 | © The Author(s).

Chronic pain[2]	HCP-reported; PwSHA's current level of chronic pain relating to their HA: None: No functional deficit; no analgesic use (except with acute haemarthrosis); Mild pain: Does not interfere with occupation nor with activities of daily living (ADL); may require occasional non-narcotic analgesic; Moderate pain: Partial or occasional interference with occupation or ADL; use of non-narcotic medications; and Severe pain: Interferes with occupation or ADL; requires frequent use of non-narcotic and narcotic medications.
Target Joints	HCP-reported; defined as per ISTH guidelines ("three or more spontaneous bleeds into a single joint within a consecutive 6-month period. Where there have been ≤ 2 bleeds into the joint within a consecutive 12-month period the joint is no longer considered a target joint")[30]. Locations include: neck, right shoulder, left shoulder, right elbow, left elbow, right wrist, left wrist, right hip, left hip, right knee, left knee, right ankle, left ankle, and spine. Subjects are also categorised into either having 0, 1, 2 or 3+ target joints.
Problem joints[5]	HCP and PwH reported; measured as any of the following symptoms: chronic joint pain, chronic synovitis, haemophilic arthropathy, limited motion or recurrent bleeding; analysed as a count of affected joints (range 0-14). Locations include neck, right shoulder, left shoulder, right elbow, left elbow, right wrist, left wrist, right hip, left hip, right knee, left knee, right ankle, left ankle, spine. Subjects are also categorised into either having 0, 1, 2 or 3+ problem joints.
On-demand FVIII infusions to treat	HCP-reported; infusions outside the normal prophylaxis treatment schedule that were used to treat an actual or suspected bleeding event.

breakthrough bleeding events	
Employment status	HCP-reported; Employed full time, employed part time, self-employed, unemployed (able to work), full and part time student, unable to work due to haemophilia, unable to work due to other reason, retired, homemaker, and other.<br>For the purposes of this analysis, categories are grouped as follows:<br>Employed; student; not employed (unemployed / unable to work due to reasons other than haemophilia / homemaker /retired); unable to work due to haemophilia; unknown.
EQ-5D	PwH reported; Health status was captured via the patient form using the EuroQol Five Dimension 5 level (EQ-5D-5L) questionnaire. The EQ-5D-5L consists of five dimensions (mobility, self-care, usual activities, pain/discomfort, and anxiety/depression) each with five levels of severity (ranging from 'no problems' to 'unable to/extreme problems'). each with five levels of severity (ranging from 'no problems' to 'unable to/extreme problems'). For this analysis, the UK EQ-5D crosswalk was applied using the mapping algorithm developed by Hernandez Alava and colleagues, allowing for observations from all countries to be assessed in aggregate.[6]
WPAI[7]	PwH reported; measured using the Work Productivity and Activity Impairment (WPAI) instrument (Reilly Associates, www.reillyassociates.net/wpaigeneral.html).The WPAI measures impairment due to a specific health problem (e.g. haemophilia A) (WPAI:SHP) during the preceding seven days.

	WPL score (expressed as a percentage) = (Q2/(Q2+Q4)) + [(1-(Q2/(Q2+Q4))) * (Q5/10)] x 100, where:<br>• Q2: Hours missed due to haemophilia;<br>• Q4: Hours actually worked;<br>• Q5: Degree to which haemophilia affected productivity while working; and<br>• Q6: Degree to which haemophilia affected regular activities
Daily activity assistance requirement	PwH-reported; reported as the need for either paid (i.e., provided by a professional caregiver) or unpaid assistance (i.e., provided by an unpaid family member or friend) for reasons relating to haemophilia.
Compromise	PwH-reported, reported using a 1-5 Likert scale to measure impact of haemophilia on specific compromise areas each reported separately. Compromise areas assessed are: social activities, exercise/physical activity, opportunities and lifestyle.
GAD-7[8]	PwH-reported; Generalized Anxiety Disorder Assessment 7 (GAD-7) is an instrument for the screening of Anxiety. Total scores of 5, 10 and 15 represent cut points for mild, moderate, and severe anxiety, respectively.
PHQ-8[9]	PwH-reported; Physical Health Questionnaire 8 (PHQ-8) is an instrument for the screening of depression. Total scores of 5, 10, 15, and 20 represent cut points for mild, moderate, moderately severe and severe depression, respectively.

Abbreviations: EHL, extended half-life; EQ-5D-5L, EuroQol Five Dimension 5 level; GAD-7, Generalized Anxiety Disorder Assessment 7; ISTH, International Society for Thrombosis and Haemostasis; PHQ-8, Physical Health Questionnaire 8; SHL, standard half-life; SHP, specific health problem; WPAI, Work Productivity and Activity Impairment; WPL, Work Productivity Loss.

Supplementary Table S2. Demographic and clinical characteristics of PwSHA in the CRF sample (n=350)

	**Emicizumab (n=191)**	**FVIII prophylaxis (n=159)**
**Age, years, mean (SD)**	34.0 (13.2)	36.1 (12.9)
**BMI, kg/m^2, mean (SD)**	24.64 (3.3)	24.80 (2.4)
**Country, n (%)**		
Italy	66 (34.5)	68 (42.8)
Spain	47 (24.6)	51 (32.1)
United Kingdom	20 (10.5)	7 (4.4)
France	26 (13.6)	13 (8.2)
Germany	32 (16.7)	20 (12.6)
**Employment, n (%)**		
Employed	131 (68.6)	116 (73.0)
Student	21 (11.0)	19 (11.9)
Not employed	19 (9.9)	15 (9.4)
Physically unable to work due to haemophilia	3 (1.6)	2 (1.3)
Unknown	17 (8.9)	7 (4.4)
**Education, n (%)**		

None or primary schooling only	1 (0.5)	14 (8.8)
Secondary, vocational, high school	114 (59.7)	83 (52.2)
University	58 (30.4)	53 (33.3)
Other or don't know	18 (9.4)	9 (5.7)
**Ethnic origin, n (%)**[a]		
White/Caucasian	152 (92.1)	139 (95.2)
Black/Afro-Caribbean	3 (1.8)	2 (1.4)
Middle Eastern	1 (0.6)	4 (2.7)
Asian-Indian subcontinent	5 (3.0)	1 (0.7)
Mixed	4 (2.4)	0 (0.0)
**FVIII treatment, n (%)**		
SHL/plasma-derived	–	76 (47.8)
EHL	–	83 (52.2)
**Previous treatment strategy, n (%)**		
Prophylaxis[b]	140 (73.3)	141 (88.7)
High-dose prophylaxis[c]	30 (21.4)	93 (66.0)
Intermediate-dose prophylaxis[d]	34 (24.3)	27 (19.2)
Other[e]	76 (54.3)	21 (14.9)
On-demand	51 (26.7)	18 (11.3)

**Treatment strategy or class switch in 12 months prior, n (%)**	54 (28.3)	25 (15.7)
**Target joints number, Mean (SD)**	0.6 (1.0)	0.5 (0.7)
**Problem joints number, Mean (SD)**	1.1 (1.5)	1.2 (1.2)
**Chronic pain, n (%)**		
None	65 (34.0)	41 (25.8)
Mild	73 (38.2)	80 (50.3)
Moderate	51 (26.7)	33 (20.8)
Severe	2 (1.0)	5 (3.1)
**ABR, mean (SD)**	1.3 (1.3)	1.2 (1.0)
**ABR in joint(s), mean (SD)**	0.6 (0.9)	0.7 (0.8)
**ABR, spontaneous bleeds, mean (SD)**	0.7 (1.0)	0.6 (0.1)
**Treated annualised ABR (≥3 months on treatment), n, mean (SD)**	n=185, 0.5 (0.9)	n=95, 0.8 (0.9)
**Annualised ABR on previous treatment, n, mean (SD)**	n=167, 2.3 (2.6)	–
**ABR difference (pre vs. post treatment), n, mean (SD)**	n=163, -1.1 (2.5)	–

Abbreviations: ABR, annual bleeding rate; EHL, extended half-life; FVIII, factor VIII; HA, haemophilia A; SD, standard deviation; SHL, short half-life.

All variables reported by HCPs unless otherwise specified.

[a]Ethnic origin data were not available for 26 patients in the emicizumab group.

[b]May include any product type (standard half-life FVIII, extended half-life FVIII, plasma-derived FVIII or emicizumab).

[c]High-dose prophylaxis defined as >4000 IU/kg/year[8].

[d]Intermediate-dose prophylaxis defined as 1500-4000 IU/kg/year[8].

[e]Comprises low-dose prophylaxis (<1500 IU/kg/year; n=21, 7.5%)[8], PwSHA previously on a different frequency of emicizumab (n=50, 17.8%), and PwSHA where the information was missing (n=26, 9.3%).
